# Ovarian Hyperstimulation Syndrome Following In-Vitro Fertilization: A Case Report

**DOI:** 10.7759/cureus.79291

**Published:** 2025-02-19

**Authors:** Jacqueline Pearlmutter, James Komara, Wayne A Martini

**Affiliations:** 1 Emergency Medicine, Creighton of Phoenix Arizona (COPA), Phoenix, USA; 2 Emergency Medicine, Mayo Clinic, Phoenix, USA

**Keywords:** ascites, emergency management, in-vitro fertilization, ovarian hyperstimulation syndrome, polycystic ovarian syndrome

## Abstract

A 34-year-old gravida 0, para 0 female with a history of in-vitro fertilization (IVF) treatment for infertility and polycystic ovarian syndrome (PCOS) presented to the emergency department (ED) with worsening abdominal pain and distention. Seven days prior, she underwent her second egg retrieval procedure. Four days post-procedure, she initially sought care in the ED, where the ultrasound revealed fluid accumulation. She opted for observation over transvaginal fluid drainage. Despite this, her symptoms progressed, prompting her to return to the ED. On physical examination, the patient exhibited abdominal distension and tenderness. Bedside ultrasound revealed free fluid in the pelvis and Morrison's pouch. Laboratory findings included leukocytosis (WBC 13.1 × 10^9/L), hyponatremia (Na 131 mmol/L), elevated C-reactive protein (19.1 mg/L), and hypoalbuminemia (3.3 g/dL). Imaging, including CT and transvaginal ultrasound, demonstrated large ascites, bilateral ovarian enlargement with theca lutein cysts, small pleural effusions, and findings consistent with ovarian hyperstimulation syndrome (OHSS). The patient underwent ultrasound-guided paracentesis in the ED, yielding 2000 mL of straw-colored fluid, which resulted in significant symptomatic relief. She was admitted for further management of moderate to severe OHSS, including fluid resuscitation and thromboprophylaxis. This case highlights the importance of early recognition and multidisciplinary management of OHSS to mitigate morbidity in patients undergoing assisted reproductive technologies.

## Introduction

Ovarian hyperstimulation syndrome (OHSS) is a rare but significant complication of assisted reproductive technologies (ART), such as in-vitro fertilization (IVF). Its pathophysiology involves an exaggerated ovarian response to controlled ovarian stimulation, leading to increased capillary permeability and subsequent fluid shifts into the third space, including the peritoneal and pleural cavities [1,2]. The incidence of moderate to severe OHSS ranges between 3% and 6% of IVF cycles, with severe forms occurring in up to 2%​​ [2,3].

Clinically, OHSS can manifest as a spectrum ranging from mild abdominal discomfort to severe complications, such as ascites, thromboembolism, and multi-organ dysfunction [3,4]. Key risk factors include polycystic ovarian syndrome (PCOS), younger age, high estradiol levels, and the use of exogenous human chorionic gonadotropin (hCG) [3]. With the increasing prevalence of ART, emergency clinicians are more likely to encounter OHSS, underscoring the importance of early diagnosis and management to reduce morbidity and mortality ​​[3,4]. This report discusses a case of moderate OHSS in a 34-year-old female, emphasizing diagnostic challenges, management strategies, and multidisciplinary approaches to optimize patient outcomes.

## Case presentation

A 34-year-old gravida 0, para 0 female presented with severe lower abdominal pain and distension following her second egg retrieval procedure for IVF. The egg retrieval was performed by her infertility specialist in Chicago, Illinois, five days prior to her emergency department visit. She reported experiencing abdominal stabbing pains starting two days post-procedure, which progressively worsened despite undergoing an ultrasound that revealed fluid buildup in the abdomen. She was offered transvaginal fluid drainage but opted for observation. Upon visiting relatives out of state, her pain became more diffuse, radiating to her back bilaterally, with associated abdominal distension but no fever, chills, dysuria, vaginal bleeding, or discharge. She had one prior IVF cycle in October 2024 that was unsuccessful.

The patient expressed concerns about abdominal distension, pain, and pelvic pain without any fever, chills, dysuria, vaginal discharge or bleeding, nausea, vomiting, or diarrhea. Her triage vital signs showed sinus tachycardia with a heart rate of 116 beats per minute. She was afebrile at 36.4°C, with a blood pressure of 127/86 mmHg, a respiratory rate of 18 breaths per minute, and a pulse oximetry reading of 98% on room air. On physical examination, she was noted to have distension with generalized tenderness with a positive fluid wave. Bedside ultrasound showed free fluid in the peritoneal sac and Morrison’s pouch. 

Out of concern for acute abdominal pathology including ectopic pregnancy, ruptured ovarian cyst, ovarian torsion, ascites due to peritoneal carcinomatosis, ovarian hyperstimulation syndrome, or pelvic inflammatory disease, a computed tomography (CT) of the abdomen and pelvis was ordered with IV (intravenous) contrast as well as a transabdominal and transvaginal ultrasound with Doppler.

**Figure 1:**
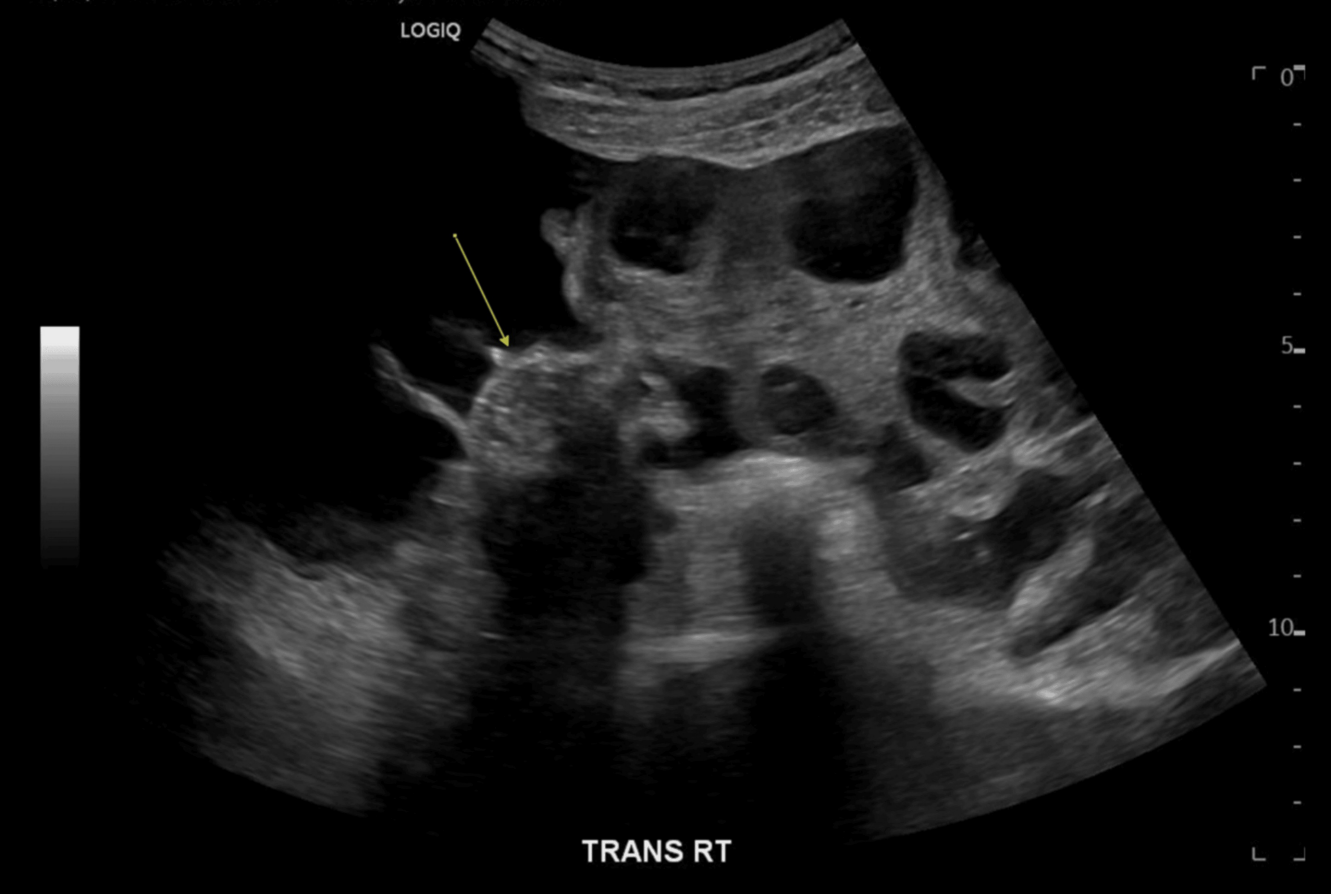
Transvaginal and transabdominal ultrasound Revealing marked ovarian enlargement (arrow) with hemorrhagic theca lutein cysts, moderate free fluid, and mild swirling of the right infundibulopelvic ligament.

**Figure 2:**
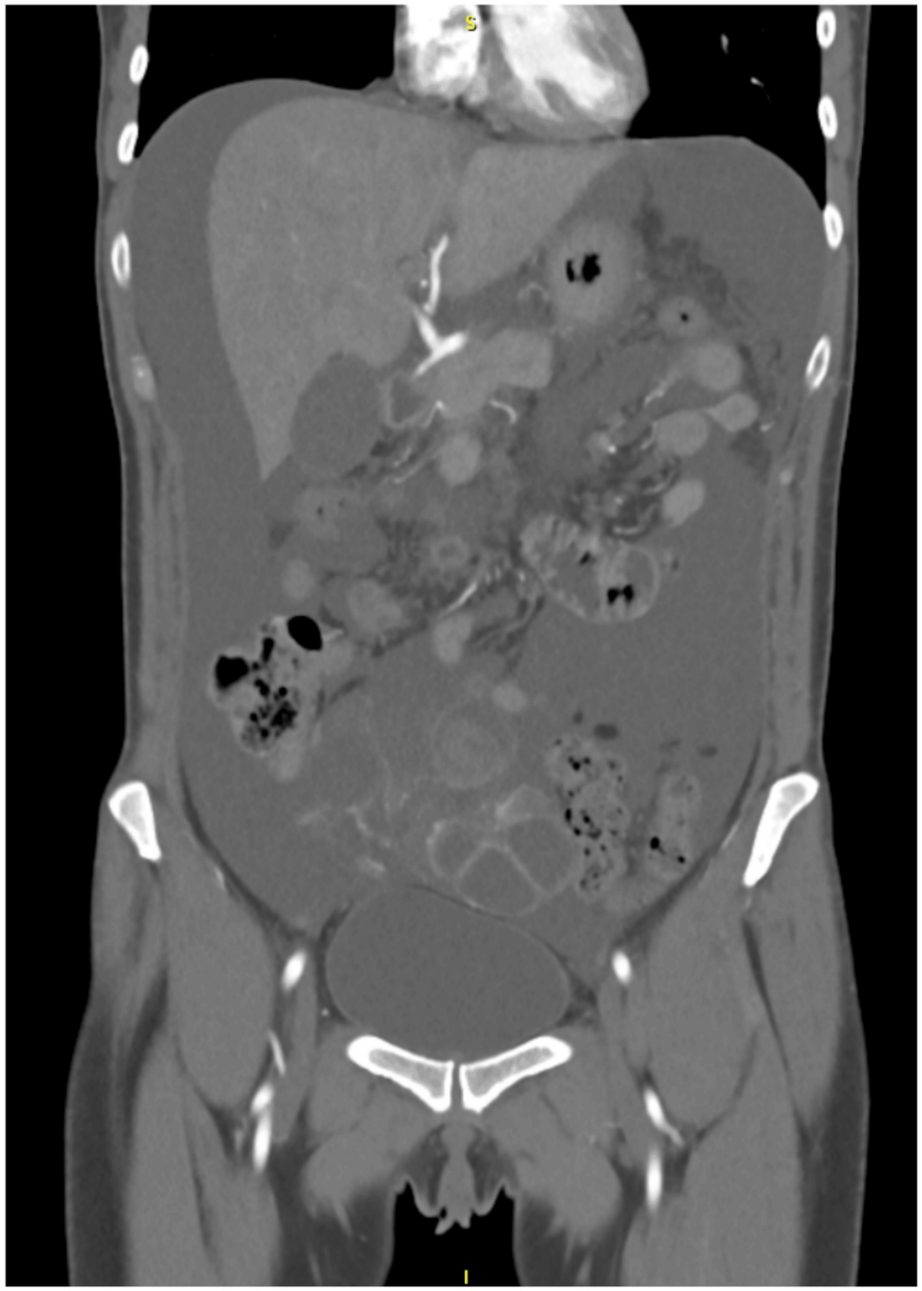
CT of the abdomen and pelvis with IV contrast Showing a large volume of ascites, small pleural effusions, and enlarged bilateral ovaries with multiple theca lutein cysts consistent with ovarian hyperstimulation syndrome.

Her complete blood count was significant for a leukocytosis of 13.1 × 10^9/L white blood cells (Table 1). A basic metabolic panel revealed hyponatremia of 131 mmol/L, hypoalbuminemia at 3.3 g/dL, C-reactive protein elevation at 19.1 mg/L, and elevated human chorionic gonadotropin (hCG) at 10.4 IU/L (Table 2), consistent with the recent Ovidrel injection. Transvaginal and transabdominal (Figure 1) ultrasound revealed marked ovarian enlargement with hemorrhagic theca lutein cysts, moderate free fluid, and mild swirling of the right infundibulopelvic ligament. CT of the abdomen and pelvis with IV contrast (Figure 2) revealed a large volume of ascites, small pleural effusions, and enlarged bilateral ovaries with multiple theca lutein cysts consistent with OHSS.

**Table 1:** Complete blood count tabulated results H: data is abnormally high; L: data is abnormally low; MCV: mean corpuscular volume

Complete Blood Count	Latest Reference Range and Units	Patient Results
Hemoglobin	11.6 - 15.0 g/dL	15.5 (H)
Hematocrit	35.5 - 44.9 %	44.5
Erythrocytes	3.92 - 5.13 x10(12)/L	5.04
MCV	78.2 - 97.9 fL	88.3
RBC distribution width	12.2 - 16.1 %	11.7 (L)
Platelet count	157 - 371 x10(9)/L	328
Leukocytes	3.4 - 9.6 x10(9)/L	13.1 (H)
Neutrophils	1.56 - 6.45 x10(9)/L	10.95 (H)
Lymphocytes	0.95 - 3.07 x10(9)/L	1.52
Monocytes	0.26 - 0.81 x10(9)/L	0.58
Eosinophils	0.03 - 0.48 x10(9)/L	0.00 (L)
Basophils	0.01 - 0.08 x10(9)/L	0.03
Nucleated RBC	/100 WBC	0.0

**Table 2:** Basic metabolic function, hepatic function panel, C-reactive protein, quantitative hCG H: data is abnormally high; L: data is abnormally low; hCG: human chorionic gonadotropin

Basic Metabolic Function, Hepatic Function Panel, C-reactive Protein, Quantitative HCG	Latest Reference Range and Units	Patient Results
Sodium, S	135 - 145 mmol/L	131 (L)
Potassium, S	3.6 - 5.2 mmol/L	4.8
Chloride, S	98 - 107 mmol/L	99
Bicarbonate, S	22 - 29 mmol/L	21 (L)
Anion gap	7 - 15	11
Blood urea nitrogen (BUN), S	6.0 - 21.0 mg/dL	12.7
Creatinine	0.59 - 1.04 mg/dL	0.86
Estimated glomerular filtration rate (eGFR)	>=60 mL/min/BSA	>90
Calcium, Total, S	8.6 - 10.0 mg/dL	8.6
Glucose, S	70 - 140 mg/dL	151 (H)
Bilirubin, Total, S	0.0 - 1.2 mg/dL	0.5
Bilirubin, Direct, S	0.0 - 0.3 mg/dL	0.2
Alanine aminotransferase (ALT), S	7 - 45 U/L	16
Aspartate aminotransferase (AST), S	8 - 43 U/L	21
Alkaline phosphatase, S	35 - 104 U/L	41
Protein, Total, S	6.3 - 7.9 g/dL	5.4 (L)
Albumin, S	3.5 - 5.0 g/dL	3.3 (L)
Lipase, S	13 - 60 U/L	17
C-reactive protein (CRP), S	<5.0 mg/L	19.1 (H)
hCG, quantitative, pregnancy, S	<5 IU/L	10.4 (H)

The patient was admitted for moderate to severe OHSS. She underwent ultrasound-guided paracentesis on the day of admission, which significantly alleviated her symptoms. Fluid resuscitation was initiated with 1 liter of isotonic saline, and serial laboratory testing showed gradual improvement in leukocytosis and sodium levels. By hospital day one, her abdominal distension and pain were markedly improved, and she tolerated a regular diet. She was discharged in stable condition with prophylactic heparin and instructions to follow up with her reproductive endocrinologist.

## Discussion

The presented case represents the moderate form of OHSS, characterized by abdominal pain, nausea, and ultrasound-confirmed ascites [1,2]. The pathophysiology of OHSS involves increased ovarian vascular permeability and fluid shifts into the third space, driven by elevated vascular endothelial growth factor (VEGF) levels. 

Patients with a predicted high response to gonadotropins based on clinical features or ovarian reserve tests should be counseled about their elevated risk of OHSS, and specific preventive measures should be considered. Due to variation between assays, it is not possible to be categorical about a threshold of serum anti-Müllerian hormone (AMH) that constitutes a high risk; however, levels above 22.5 pmol/l have been shown to have an optimum balance of sensitivity and specificity [5-7]. A higher AMH threshold is associated with greater specificity but lower sensitivity. Antral follicle count (AFC) above 20 has been associated with an increased risk of OHSS [5-7]. 

Several studies have identified polycystic ovarian morphology on ultrasound as well as PCOS as risk factors for OHSS [8-11]. A meta-analysis of 11 studies identified a significantly higher risk of OHSS in women with PCOS compared to women without this diagnosis [12]. Ovarian hyperstimulation syndrome necessitates careful monitoring due to its potential progression to severe stages, which involve life-threatening complications such as acute respiratory distress syndrome, thromboembolic events, or renal dysfunction [3,4].

The diagnosis of OHSS hinges on clinical suspicion supported by imaging and laboratory findings. Ultrasound serves as the cornerstone for identifying ovarian enlargement and ascites, with point-of-care ultrasound enabling prompt recognition in emergency settings [3,13]. Laboratory tests, including hematocrit and serum electrolytes, are critical for assessing hemoconcentration and electrolyte imbalances [3].

Continuous evaluation of vital signs, fluid balance, and laboratory parameters is essential. Admission is often warranted for moderate cases to monitor for worsening symptoms or complications [2,4]. Initial fluid resuscitation with isotonic saline aims to restore intravascular volume. Serial monitoring of electrolytes and hemodynamics guides further fluid therapy​​ [3,4]. Paracentesis can alleviate symptoms of ascites and reduce intra-abdominal pressure, improving respiratory and renal function​​ [2,3]. Prophylactic anticoagulation should be considered, especially in patients with significant hemoconcentration or other thrombotic risk factors​​ [3,4]. Coordination with the patient's reproductive endocrinologist is crucial for individualized care and planning future assisted reproductive technology (ART) cycles​​ [2,13].

## Conclusions

This case report underscores the pivotal role of emergency clinicians in the early recognition and management of OHSS, a potentially life-threatening complication of assisted reproductive technologies. As the use of assisted reproductive technologies continues to rise, clinicians must maintain a high index of suspicion for OHSS in women presenting with unexplained abdominal pain, ascites, or a recent ART history. Early diagnosis through clinical suspicion, imaging, and laboratory findings, combined with prompt targeted interventions such as ultrasound-guided paracentesis and thromboprophylaxis, is essential to mitigate morbidity and improve patient safety. Effective multidisciplinary collaboration is instrumental in optimizing outcomes and guiding future reproductive care. Educational initiatives and clinical guidelines can further enhance awareness and preparedness for OHSS among emergency providers, ensuring improved patient outcomes and safety.
